# The Evolution and Integration of Life and Theory in Foucault’s Work on Power

**DOI:** 10.1007/s12124-024-09862-8

**Published:** 2024-08-14

**Authors:** Line Joranger

**Affiliations:** Faculty of Health and Social Sciences, Department of Health, Social and Welfare Studies, University of South-Eastern Norway, Vestfold, Norway

**Keywords:** Foucault, Power, Life experiences, Collective experiences, Culture, Evolution, Theory, Revolution, Integration, Ideas

## Abstract

The article is a response to Kaldybekov and his colleague’s, 2024 paper about Foucault’s theory on power. I argue that it is difficult to understand Foucault’s theory of power without looking into his intellectual life and experiences, especially his war experiences. The objective of my study is to show that there is a connection between Foucault’s ideas about power and his own lived life, and that he always has been critical of totalitarian theories although he seems influenced by Marxist theories, early in his career. In the paper I show how he deals with this dilemma by incorporating some of Nietzsche’s ideas into his thinking. To illustrate the connection between Foucault’s lived life and his theories about power, I take a particular point of departure in Foucault’s lecture series on psychiatric power in the 1970s and an interview conducted by the Italian journalist Trombadori.

In the article “Problem of Power in Michel Foucault’s Philosophy” Kaldybekov and his colleagues present what they call the French historian and philosopher Michel Foucault’s innovative ideas about power (Kaldybekov, Y., Abdildin, Z., Kabul, O., & Tumashbay, T. (2024). Problem of Power in Michel Foucault’s Philosophy. *Integr Psychol Behav Sci*, *58*(2), 420–432. 10.1007/s12124-023-09804-w.). They state that Foucault’s works are significant for modern researchers in various branches of science since he created a universal tool for the study of social relations. The objective of their study entails conducting a thorough examination of the development of his theory, as well as exploring diverse perspectives presented by scholars on this matter. The main conclusion of the paper is that ‘power’ is a complex social phenomenon that cannot be equated with categories such as ’law’, ’discipline”, ‘subordination’, or ‘punishment’.

In this article I argue that it is difficult to understand Foucault’s theory about power without looking into his intellectual life and experiences, especially his war experiences. The objective of my study is to show that there is a connection between Foucault ideas about power and his life experiences. I also show that Foucault, through his career, was sceptical to totalitarian theories, although he seems influenced by a time typical Marxism. In the paper I show how he escapes this dilemma by incorporating some of Nietzsche’s ideas to his Marxist thinking. To illustrate the connection between Foucault’s lived life and his theories about power, I take a particular point of departure in Foucault’s lecture series on psychiatric power in the 1970s and an interview conducted by the Italian journalist Trombadori.

## Marx’s Early Influence on Foucault and his Contemporaries

As a psychology and philosophy student in the 40s and 50s Foucault and his fellow students were introduced to the ideas of Marxism at the École Normale Supérieure. That is, like his fellow students, he allowed himself to be influenced by both Nietzsche’s and Marx’s ideas. At the urging of his Marxist-oriented teacher, Louis Althusser, he also joined, if somewhat doubtfully, the French Communist Party: Parti communiste francais (PCF). He explains it this way: “I decided to join the Communist party. This was in 1950. I was a Nietzschean Communist! To be a ‘Nietzschean Communist’ is something really close to impossible to live with and even a little ridiculous, if you like. Even I knew it was” (Foucault, in Eribon, 1991, p. 52).

In a 1978 interview with the Italian Marxist journalist, Duccio Trombadori that was later translated into English with the title, *Remarks on Marx*, in 1991, and then reappeared in a new translation with the chapter title, “An Interview with Michel Foucault” in *Power: Essential Works of* Foucault, 1954*–1984* (2000, p. 281), Foucault continues:

For many of us, young intellectuals, interest in Nietzsche (…) did not represent a way of distancing oneself from Marxism or communism. On the contrary, it was the sole route of communication, the only way to get through to what we thought we should expect from communism. This requirement that we totally reject the world in which we had had to live was certainly not satisfied by Hegelian philosophy. On the other hand, we were also seeking other intellectual routes to reach precisely that point at which something entirely different seems to take shape or exist: that is communism. (Foucault & Trombadori, 1991, pp. 50–51)

Althusser becomes one of the leading figures in what was to become the French Marxist-oriented structuralist movement. His structuralist reading of Marx centred largely on Marx’s later writings, *Grundrisse and Das Kapital*. It took Marx as a scientist as a point of departure, i.e. how he in these texts documented the economic-materialist determinism that manifested itself in social development (Schrift, 2006, p. 87).

Althusser’s sharp distinction between what he considered ideology and science, fiction and fact, or what he also saw as the early Hegelian Marx, and the later scientific Marx, was strongly influenced by the French epistemologist Gaston Bachelard’s scientific attempt to unify the human sciences and scientific paradigms in a unified science of man and society. He wanted to show his contemporaries the scientific epistemology that he believed both Marx and Engels represented, if somewhat unconsciously, through their theories (Smart, 1983, p. 16).

Like the ethnologists who wanted to show that humans were not subjects of their (symbolic) actions, Bachelard, like Marx, wanted to illuminate that humans were not subjects of their history. He wanted to develop a Marxist theory that could work in opposition to the humanist Marxism, which he believed that the contemporary French philosophers Jean-Paul Sartre and Maurice Merleau-Ponty represented. In contrast to Sartre’s and Merleau-Ponty’s existentialist ideas which saw the individuals as an active participant in shaping their world with others (the social world), Althusser saw Marxism as both anti-humanist and deterministic:

Marx’s theoretical anti-humanism, as it operates within historical materialism, refers to a resistance to explaining the social formations and their history with a starting point in theoretical concepts, i.e. concepts that man is a product of origin that connects to his needs (homo oeconomicus), his thoughts (homo rationalis), and his struggles and actions (homo moralis, juridicus and politicus). (Althusser, 1976, p. 205)

We can hear the echo of Althusser’s Marxist and anti-humanist ideas in Foucault’s lecture series on psychiatric power in the 1970s. At first glance, it may seem that Foucault, like Marxist structuralism, describes the relationship within the first psychiatric treatment scenes as a relationship overridden by a historical materialism, and an asymmetrical power relationship between ruler and subject. It may seem that Foucault, like Marx’s historical and materialistic analyses of the power of the elite (the ruler) over the propertyless workers (subjects), will show how the power of modern psychiatry grows out of a specific historically hierarchical power into the age of a more subtle more or less ruler-less disciplinary power (Foucault, 2003a, pp. 43–44).

## The Revolutionary and Liberation Movements Impact on Foucault’s Power Perspectives

Revolutionary and liberation movements of the late 1960s and in the 1970s were the first great attempt to confront the structures of power that still rule over us today. In some respects, we can recognize in those movements the seeds of practices, concepts, and political aspirations that have developed since; but in other respects, the movements of the 1970s were ahead of us and we need to catch up to them. Foucault gives his lectures on psychiatric power in a period where both he and his listeners are influenced by the revolutionary events that took place during the student uprisings in Paris in the spring of 1968. Foucault is also influenced by the war experiences he acquired while living in Tunisia in the period between 1966 and 1968. During this period, Tunisia was hit by several very violent political demonstrations directed at the state of Israel, and Foucault gained important experience with military regimes as a political activist. During his stay in Tunisia, he also wrote the book *The Archaeology of Knowledge* (Foucault, 1969; English translation, 1972) (Foucault, 1972). Here Foucault analyses the use and formation of discourses, which we find again both in the psychiatry lecture and in the lecture on discourses from 1970: “The Order of Discourse” (“L’ordre du discours”) (Foucault, 1971). In both lectures, Foucault talks about the discipline as a separate system of exclusion within the modern knowledge society.

To Foucault, the discipline is defined by an object area or a knowledge area, such as psychiatry and medicine. It is characterized by a set of methods and rules and a collection of true statements. Its techniques and aids constitute a kind of anonymous system that is available to anyone who wants to or can use it without its validity being linked to the person who invented it. It is also known that during his psychiatry lectures Foucault worked on the book *Discipline and Punish: The birth of the prison* (Surveiller et punir) (Foucault, 1977a). It was in this book that the concept of punishment and knowledge (punir et savoir) was developed during the discussions on forensic psychiatry. In the lecture series “Society must be defended” (Il faut défendre la société) (Foucault, 2003b), which Foucault gave at the Collège de France in 1976, one can also see how in many ways he transfers his military conceptual apparatus from the psychiatry lectures to his descriptions of how the modern society establishes a need to protect and control its political interests in a way that requires one to go to “war” against the uncontrollable and unknown. As in the psychiatry lecture, in “Society must be defended” there is talk of an anonymous and classless power, established in an impersonal network that cannot be easily located either here or there.

If we look more closely at Foucault’s life and work in the early 1970s, a couple of years after the May Revolution he became heavily involved in the militant French Information Group GIP, *Groupe d’Information sur les prisons*, or *Gauche Prolétarienne*, as it was also called. It consisted, among other things, of a multitude of self-philosophy and the impact of experience on Foucault’s analysis created Maoists, with the aim of providing legal information to inmates in French prisons, in addition to their own militant actions (Eribon, 1991; Miller, 1993, pp. 176–177). The group was later succeeded by another group, GIA: *Group d’information sur les asiles*, which had the asylum as its starting point.

On the basis of his experiences from Tunisia, where the activists were far more exposed to punitive reactions than their activist brothers in France, it seems that Foucault, like the Maoists, wanted to experiment with different types of political actions which meant that he had to get involved both physically and psychologically (Foucault, 2000, pp. 239–297). By involve himself this way, it seems that Foucault recaptured what had preoccupied him when he worked with madness, and what he had just experienced in Tunisia.

In the above mentioned interview with Trombadori (2000, p. 281), Foucault says:

(…) when I returned to France in November or December of 1968, I was surprised, astonished, and even disappointed, considering what I had seen in Tunisia. In spite of their violence, their passion, the struggle had not involved the same cost, the same sacrifices (…). People in France spoke of hyper-Marxism, of a proliferation of theories, of a splintering into small groups. It was exactly the opposite, the reverse, the contrary of what had intrigued me in Tunisia. That may explain the way in which I tried to approach things from that time onward, away from those endless discussions, that hyper-Marxization, that irrepressible discursivity which characterized university life (…) I tried to do things that required a personal, physical, and real involvement (…). It was only from that moment that necessary analyses could be proposed. Working with the GIP on the problem of the prisoners, I attempted to initiate and carry through an experience. At the same time, it also gave a kind of occasion for me to revisit what I had been concerned with in works like *Madness and Civilization* or *The Birth of the Clinic* and to reflect on what I had just experienced in Tunisia.

## A Philosophical Journey between Marxism’s and Nietzsche’s Understanding of Power

In his lecture series on psychiatric power in the 1970s Foucault makes it clear that his new analyses represent a military field of experience. He admits in one of his interviews with Trombadori that without the May Revolution he would never have been able to analyse either the prison system or the power game that was going on inside the modern psychiatric institutions in the way he came to do in the 1970s (Foucault, 2000, p. 282). After the revolution he had begun to question the underlying political causes of the discontent and uprisings in Tunisia and France, and the rest of the world. What was everyone questioning? According to Foucault, the question everyone asked represents a common feeling of permanent oppression maintained by a state apparatus, by different groups, and or different institutions. People no longer wanted to be led, they no longer found themselves subject to power. From all these experiences only one word emerged, Foucault says, including his own experience: “power” (Foucault, 2000, p. 284). In the interview with Trombadori, Foucault explains that he in retrospect sees that what he previously wrote about the genealogy of knowledge history was about power. He admits that his previous analysis of reason and unreasonableness, right and wrong, and normalities, was a single large story of the genealogy of power, or what he himself calls the history of power.

As previously mentioned, Foucault may have been inspired by the revolutionary political ideas of Marxism when he developed his theories about power and gave his lecture series “Psychiatric Power”. However, if we look more closely, we can also find Nietzsche’s ideas in his works and lectures about power. There are several factors that speak for this. The first is that Foucault constantly seems to be critical of universal and global theories about humans and society. The second is that he does not use the genealogy to criticize or promote a singular program, a prophecy, or a political theory, as traditional Marxism did. Like Nietzsche, Foucault’s analyses seem to be situated in a space beyond descriptions of ‘good’ and ‘bad’, and ‘right’ and ‘wrong’. He himself says that criticism should not be the premise for a deduction that concludes (Baynes et al., 1987, p. 114). According to Foucault, criticism should be an instrument for those who fight, and for those who oppose what is, whether it is Marxism, Maoism or capitalism: “It needs not to lay down the law for the law. It is not a phase in a program. It is a challenge aimed at what is’ (Baynes et al., 1987, p. 114).

The third relationship that indicates that Foucault may have derived his perspectives from Nietzsche is his notion of Western humanism. In an interview from 1971, he explains in Nietzschean terms that Western humanism is aimed at limiting the individual’s “will to power” (Foucault, 1977b, pp. 221–222). This means that all attempts to create a self, and all sensations of an ‘I’, are erased out in favour of a socially constructed person who has learned to conform to what is. Understood in this way, both doctor and patient, in Foucault’s examples of psychiatry, are physically and psychologically programmed to conform to the structures within which they find themselves. They are both oppressed in their role, and they both fight a battle to mark an authentic ‘I’.

In The *Will to Power* (Der Wille zur Macht) Nietzsche uses the term “will to power” to describe the self’s striving to become an authentic ‘I’ (Nietzsche et al., 1968, No. 1003). More than describing power in relation to others, acts that for Nietzsche of using one’s will to effectuate one’s higher order preferences. In the book Nietzsche: Life as Literature, Alexander Nehamas writes in the chapter “How one becomes what one is”, that for Nietzsche it is not only about “becoming who you are”, it is not only about improving yourself, it is about more radical about becoming (Nehamas, 1985). Similar to Foucault’s descriptions of how the power of psychiatry takes shape during the industrial society’s need for control of the human psyche and behavior, Nietzsche describes in *The Guy Science* (*Die Fröhlichen Wissenschaft*) how Western industrialism comes to suppress the specific the will to authenticity by turning it against industrial society’s collective need for loyalty and worship of the community’s goods (Nietzsche, 1974, p. 92). What Foucault describes as an anonymous, nameless, disciplinary micro-power within the first scenes of psychiatry, Nietzsche describes in *The Guy Science* as a modern tendency to in a subtle way bring all the unknown back to the known, and the uncontrollable back to the familiar and controllable (Nietzsche, 1974, p. 300).

If we go back to the beginning of the 1950s, i.e. the period when French Marxism fully established itself, it is interesting to note that already in Foucault’s first two texts from 1954: *Mental Illness and Personality* (Maladie mentale et personnalité) (Foucault, 1954), and the introduction to the French translation of the German-Swiss psychiatrist Ludwig Binswanger’s essay *Dream and Existence* (Traum und Existenz) (Binswanger, 1954), or *Le Rêve et l’Existence* as it came to be called in French, Foucault seems to distance himself from radical Marxism and Althusser’s ‘scientific’ determinism and anti-humanism, although several Foucault analysts, including O’Farrel, seem to think that it is precisely these perspectives he describes in his first texts (O’Farrell, 2005, p. 25).

In his 1954 texts, Foucault seems to turn his gaze more in the direction of the Marxist existentialism and the Marxist phenomenology, that is, the same Marxist approach that we find in Sartre and Merleau-Ponty’s work at the time. Like them, Foucault in his 1954 texts is concerned with the development of consciousness in the creative self. In contrast to revolutionary Marxism and to Althusser’s structuralist project, Foucault, 1954 project is to show that the subject is precisely a subject of his or her history. According to Foucault, a patient’s disease should not be sought in a specific evolution, but in the patient’s individual history and life conditions.

Foucault ends his first book, *Mental Illness and Personality*, with a quote that shows that he in 1954, like Sartre and Merleau-Ponty, among others, also fought for the ideas of humanism, which he later distanced himself from:

The true psychology must get rid of the abstractions, which help to separate the psychological aspects of the disease from its life conditions, and thus hide the actual causes of the disease and the reality of alienation. When it comes to man, abstraction is not merely an intellectual error: true psychology must rid itself of psychologism, if it is right that it, like all human sciences, should aim at liberation. (Foucault, 1954, p. 110)

Foucault’s aversions to totalitarian theories and various isms seems to be charactering his thinking throughout his career and writings. His obvious scepticism towards totalitarian theories shows that Foucault, even though his intellectual environment was influenced by Marx’s political perspectives, is not entirely Marxist, nor is he entirely Nietzschean, because Nietzsche never linked his perspectives to military experiences. In many ways, as he himself says, he is a mixture of both Marx and Nietzsche. Although Foucault is not alone in experiencing the intervention of military power during the revolutionary riots of 1968, his work shows that he received and processed his experiences in different ways. Compared to other contemporary thinkers who obviously must have experienced much of the same as Foucault in the 1960s, such as philosophers as Martin Heidegger, Jürgen Habermas, Sartre, Simone de Beauvoir, Merleau-Ponty, Pierre Bourdieu and many others, none of these thinkers in the decade after the 1968 revolution chooses to dedicate their entire intellectual career to the perspective of power and a military conceptual apparatus, as Foucault did. Like Foucault, they did adapt a socially critical way of thinking. Although Foucault’s experiences are time typical, they also connect to his unique personal experience and to the actual events that he attended and reflected up on. This made him to a unique contemporary writer and thinker.

What contemporary French and German thinkers collectively experienced as the control and disciplining systems of military power in the first half of the 1940s and at the end of the 1960s contributed, Foucault transformed into a new reading of both the history of madness, the history of psychiatry and the history of political philosophy. His personal encounter with military power in 1968 signalled in a different way than before what he considered important and less important in the 1970s. It also led him away from the moral perspectives in the *History of Madness*. Nevertheless, in his lectures on the psychiatric power in the 1970s, Foucault seems to be aware that there are several worldviews and knowledge areas and what these looked like. By exchanging concepts from shared cultural and military experiences, he also manages to convey some unique thoughts that he was initially alone of.

During his first lecture on psychiatric power, Foucault makes it clear that his previous book, *History of Madness*, must be seen as the start of his psychiatry lecture series at the Collage de France, even though he has undergone major theoretical and ideological changes over the years (Foucault, 2003a, p. 14). He explains that he during his lectures will correct what he perceives as weaknesses in the *History of Madness*. This particularly applies to the last chapter where he discusses institutional power (Pouvoir asilaire) (Foucault, 2003a, p. 14). Foucault’s new theoretical and ideological changes seem to challenge him methodically and lead him to develop new perspectives that breaks or extends his earlier perspectives in *History of Madness*. He wants to continue from an analysis of representations to an analysis of power, and from an analysis of violence to an analysis of the microphysics of power, and from an analysis of institutional regularity to “dispositions” du pouvoir (Foucault, 2003a, pp. 14–18).

Foucault gives his series of lectures on “psychiatric power” (Le pouvoir psychiatrique) at the Collège de France, between 07 November 1973 and 06 February 1974. He looks at early nineteenth century psychiatric treatments praxis more than 150 years after they have taken place. He presents them to his listeners as examples that support his analysis. He wants to reinterpret the early psychiatric treatment praxis with the aid of military concepts (Joranger, 2013). His project is historical; through a genealogical approach, he wants to show that modern psychiatric practice is built upon specific historical power structures and needs (Joranger, 2013). In his second lecture he states:

What I would like to do this year is basically a history of these psychiatric scenes (…) the game of power which is sketched out in it, should be analysed before any institutional organisation, or discourse of truth, or importation of models (…). It seems to me that if we want to produce a true history of psychiatry (…) it will be by situating it in this series of scenes - scenes of the ceremony of sovereignty, of rituals of service, of judicial procedures, and of medical practices - and not by making analysis of the institution the essential point and our point of departure. Let’s be really anti-institutionalist. What I propose to bring to light this year is, before analysis of the institution, the microphysics of power (Foucault, 2003a, pp. 33–34).

Foucault states in his opening lecture that he has derived his concepts from a particular field of knowledge. He explains that he wants to avoid using concepts from sociology and psychology, since this would put him on the wrong track and hinder him from describing psychiatric power as it manifested itself in the eighteenth and nineteenth centuries (Joranger, 2013, p. 3). It is not until later that he sees he has instead used terms from a pseudo-military field of knowledge in his opening lecture.

He tells us that even whilst preparing his lecture notes, he had not been aware of which field of knowledge he was drawing his concepts from. Foucault points to examples in his manuscript that he now realises are not described as theatrical episodes but as ‘rituals’, ‘strategies’ and ‘battles’. This terminology means that his themes are expressed in terms of war and military models (Joranger, 2013, p. 3).

Foucault’s new focus in the early 1970s concentrates around questions concerning the relations of power to discursive practices, i.e. more about external immediate conditions than about internal mechanisms. His earlier concepts of ‘representation’, ‘violence’ and ‘institution’ no longer function fully as explanatory models for what he now sees as an historically important political and psychological power mechanisms, that spread itself at all layers of the society (Joranger, 2013). Foucault seems to have turned his gaze away from the domains’ internal affairs towards the immediate power mechanisms outside the representations themselves, the violence, and the institutions. In his psychiatric power lectures, he explains this transition as a transition between what he now sees as two historical forms of power, that is the explicit, physical, and hierarchical ruling power that we find again in the post-feudal and pre-industrial society, and the disciplinary micro-power found in the subsequent industrial society (Joranger, 2013).

Foucault changes his concepts and understanding of power through his life and career. In the 1970s his understanding of power and his use of military concepts seems to become the bridge that connects his thoughts and experiences to a contemporary understanding of war and revolution. By examining his work and his life experiences we can see how life and theory integrate and evolve in Foucault’s work on power. In an interview from 1980, he explains: “I never think exactly the same because my books constitute experiences, in the broadest sense of the word. An experience is something that you leave changed. (…) I am an experimenter in the sense that I write to change myself, and not to think the same as before” (Foucault, 2001, pp. 860–861).

## Data Availability

No datasets were generated or analysed during the current study.
